# Low rates of liver injury in edoxaban users: Evidence from a territory‐wide observational cohort study

**DOI:** 10.1002/clc.23570

**Published:** 2021-02-16

**Authors:** Jiandong Zhou, Keith Sai Kit Leung, Dicken Kong, Sharen Lee, Tong Liu, Abraham Ka Chung Wai, Carlin Chang, Qingpeng Zhang, Gary Tse

**Affiliations:** ^1^ School of Data Science City University of Hong Kong Hong Kong China; ^2^ Aston Medical School Aston University Birmingham UK; ^3^ Cardiovascular Analytics Group Laboratory of Cardiovascular Physiology Hong Kong China; ^4^ Li Ka Shing Institute of Health Sciences The Chinese University of Hong Kong Hong Kong China; ^5^ Tianjin Key Laboratory of Ionic‐Molecular Function of Cardiovascular Disease, Department of Cardiology, Tianjin Institute of Cardiology Second Hospital of Tianjin Medical University Tianjin China; ^6^ Emergency Medicine Unit, Li Ka Shing Faculty of Medicine The University of Hong Kong Hong Kong China; ^7^ Division of Neurology, Department of Medicine Queen Mary Hospital Hong Kong China

Dear Editor,

We read the recent meta‐analysis by Dai et al. on liver injury caused by oral anticoagulants with great interest.^1^ With the widespread use of direct oral anticoagulants (DOACs) in clinical practice for the prevention of thrombotic events,^2^, ^3^ certain concerns have been raised with its risk of inducing liver injury. Such findings were not revealed in clinical trials, with a meta‐analysis demonstrating no significant associations between DOAC use and liver injury.^4^ Specifically regarding edoxaban, the landmark ENGAGE AF‐TIMI 48 trial found that hepatic events, as defined by AST or ALT ≥3× upper limit of normal or above with total bilirubin ≥2× upper limit of normal, were comparable among the warfarin, high‐dose and low‐dose edoxaban groups.^5^ However, the occurrence of drug‐induced liver injury for DOACs has been reported by several case reports and series in post‐market settings, and has been summarized by Liakoni et al. as early in 2015.^6^ These variations in results have led to investigations of drug‐induced liver injury by individual DOACs in observational studies.^7^, ^8^, ^9^, ^10^, ^11^ Nevertheless, limited data are available for edoxaban, given its comparative late entry into the international market. Therefore, we conducted this territory‐wide study to assess the risk of liver injury in edoxaban users who have no prior history of liver diseases. This study received approvals from Institutional Review Board of the University of Hong Kong—Hospital Authority Hong Kong West Cluster, and the Joint Chinese University of Hong Kong—Hospital Authority New Territories East Cluster Clinical Research Ethics Committee. The detailed methods are shown in the **Supplementary** Appendix.

Among the 2107 edoxaban users from January 01, 2016 to December 31, 2019 initially identified, 894 patients were excluded for the following reasons: (1) prescriptions of other anticoagulants (*n* = 543); a prior diagnosis of liver disease (*n* = 16); prior liver injury (*n* = 335) (Figure 1). Finally, 1213 edoxaban users were included (Table 1, left), with 19 developing liver injury after its prescription, which corresponded to an incidence of 1.5% (Figure 2). Univariate Cox regression identified significant predictors of liver injury (Table 1, right), which were: older age (HR: 1.32, 95% CI: [1.03, 3.29], *p* = .006), prior hemorrhagic stroke (HR: 5.24, 95% CI: [1.53, 17.99], *p* = .009), and higher levels of creatinine (HR: 1.002, 95% CI: [1.000, 1.005], *p* = .021) and urea (HR: 1.10, 95% CI: [1.04, 1.16], *p* = .001).

**FIGURE 1:**
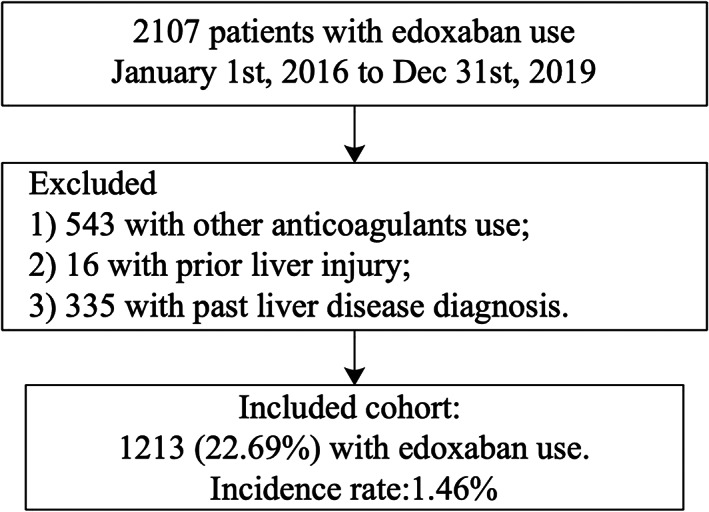
Procedures of data processing

**TABLE 1:** Baseline characteristics of edoxaban users and Cox regression results for significant predictors of liver injury

Characteristics	Edoxaban (*N* = 1213) median (IQR); max; N or count (%)	HR (95% CI)	*p*‐value
Demographics			
Baseline age, year	74 (64–82); 100; *n* = 1213	0.99 (0.96, 1.02)	.584
Male gender	622 (51.27%)	1.32 (1.03, 3.29)	.006**
Past comorbidities		0.91 (0.33, 2.53)	.856
Respiratory	345 (28.44%)	1.04 (0.30, 3.56)	.955
Endocrine	18 (1.48%)	1.65 (0.38, 7.15)	.502
Diabetes mellitus	84 (6.92%)	0.67 (0.24, 1.87)	.449
Hypertension	440 (36.27%)	0.72 (0.17, 3.11)	.657
Gastrointestinal	170 (14.01%)	0.70 (0.16, 3.02)	.630
Hemorrhagic stroke	15 (1.23%)	5.24 (1.53, 17.99)	.009**
Congestive heart failure	28 (2.30%)	2.72 (0.36, 20.41)	.330
Gastrointestinal bleeding	86 (7.08%)	0.70 (0.09, 5.26)	.731
Medications			
Edoxaban duration, day	313 (236–424); 228; *n* = 1213	0.69 (0.67, 0.73)	.002**
ACEI	422 (34.78%)	0.93 (0.35, 2.44)	.874
ARB	222 (18.30%)	0.57 (0.13, 2.49)	.459
Calcium channel blockers	665 (54.82%)	0.51 (0.20, 1.30)	.160
Beta blockers	625 (51.52%)	0.75 (0.30, 1.86)	.531
Diuretics for heart failure	512 (42.20%)	1.73 (0.70, 4.28)	.232
Nitrates	272 (22.42%)	0.68 (0.20, 2.32)	.534
Antihypertensive drugs	231 (19.04%)	0.51 (0.12, 2.22)	.372
Statins and fibrates	654 (53.91%)	0.54 (0.21, 1.37)	.191
Antihyperlipidemic/Lipid‐lowering drugs	646 (53.25%)	0.55 (0.22, 1.41)	.216
Laboratory tests			
Creatinine, umol/L	87 (71–111); 1192; *n* = 1085	1.002 (1.000, 1.005)	.021*
Urea, mmol/L	6.1 (5–8); 40.6; *n* = 1084	1.10 (1.04, 1.16)	.001***
Potassium, mmol/L	4.1 (3.8–4.4); 6; *n* = 1085	0.70 (0.25, 1.91)	.482
Sodium, mmol/L	140 (138–142); 155; *n* = 1085	0.93 (0.81, 1.08)	.350
Urate, mmol/L	0.41 (0.3365–0.521); 0.98; *n* = 207	19.51 (0.06, 6018.00)	.310
Albumin, g/L	39 (34.6–42); 51.7; *n* = 937	0.94 (0.87, 1.01)	.077
Protein, g/L	72 (67–76); 92.48; *n* = 882	0.99 (0.93, 1.06)	.817
Alkaline phosphatase, U/L	74.55 (61–92); 454; *n* = 936	1.00 (0.98, 1.01)	.818

* *p* ≤ .05.

** *p* ≤ .01.

*** *p* ≤ .001.

**FIGURE 2:**
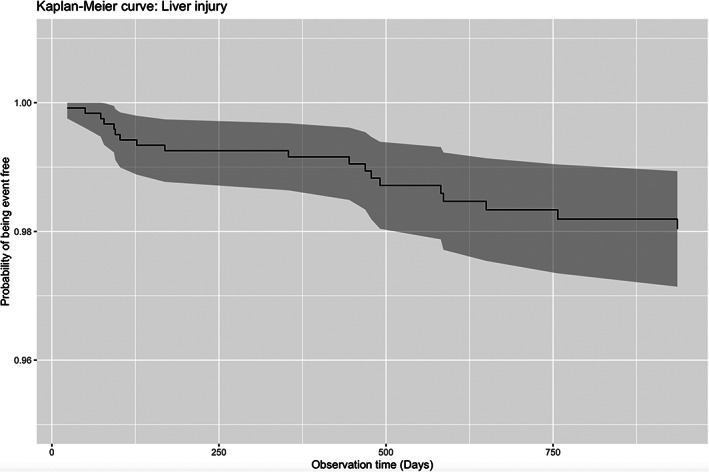
Kaplan–Meier curve of liver injury in edoxaban users

The main finding of this study is that there is a low rate of liver injury of 1.5% in patients receiving edoxaban therapy in Hong Kong. This extends previous findings by another local study, which reported an incidence of 1.8% for dabigatran, 2.1% for rivaroxaban, 2.5% for apixaban, with a pooled incidence rate of 2.0% for all three DOACs, as compared to 3.7% for warfarin.^8^ Edoxaban, similar to the other DOACs, has demonstrated at least non‐inferiority compared to warfarin for stroke prevention in patients with atrial fibrillation.^12^, ^13^, ^14^ Our data provide further evidence regarding the safety of edoxaban specifically regarding hepatotoxicity. However, significant factors include advancing age, impaired renal function and prior haemorrhagic stroke. Therefore, this medication should be used in caution in such users, who should receive regular blood tests. Our findings should be validated in future observational cohort studies or clinical trials. Moreover, a follow‐up analysis of the ENGAGE AF‐TIMI 48 trial demonstrated that a history of liver disease did not significant affect the efficacy of safety of edoxaban compared to warfarin and the rates of hepatic adverse events were similar between edoxaban or warfarin users.^15^ Future observational studies should examine the efficacy and safety outcomes in this specific subgroup to complement findings from this important trial.

## CONFLICT OF INTEREST

None.

## Supporting information


**Appendix** S1. Supporting Information

## Data Availability

The data described in this letter are available upon request to the corresponding authors.
