# Supplementary material for: Low rates of liver injury in edoxaban users: Evidence from a territory‐wide observational cohort study
Source: Clin Cardiol. 2021 Feb 16;44(7):886–9. doi: 10.1002/clc.23570 (PMC8259145; doi:10.1002/clc.23570)
Supplement: Supplementary file 1 — Appendix S1. Supporting Information [file CLC-44-886-s001.docx]

**Supplementary Appendix**

**Methods**

This was a retrospective, territory-wide cohort study of patients between January 1^st^, 2016 and Dec 31^st^, 2019 in Hong Kong. Patients who developed liver injury, were prescribed with anticoagulants other than edoxaban, or had diagnoses of liver diseases were excluded. Liver injury was defined as the earliest occurrence of an alanine aminotransferase (ALT) or an aspartate aminotransferase (AST) serum level greater than 3 times the upper limit of normal, (ULN) and a total bilirubin level greater than 2 times the ULN. ULN for AST is 38 U/L, ULN for ALT is 50 U/L, ULN for T-BIL is 22 umol/L.

The patients were identified from the Clinical Data Analysis and Reporting System (CDARS), a territory-wide database that centralizes patient information from individual local hospitals to establish comprehensive medical data, including clinical characteristics, disease diagnosis, laboratory results, and drug treatment details. Mortality data were obtained from the Hong Kong Death Registry, a population-based official government registry with the registered death records of all Hong Kong citizens linked to CDARS. The system has been previously used by both our team and other teams in Hong Kong (10). Patients demographics, prior comorbidities, medication prescriptions, laboratory examinations of creatinine, urea, potassium, sodium, urate, albumin, protein, alkaline phosphatase, aspartate transaminase, alanine transaminase, and total bilirubin were extracted. The list of ICD-9 codes for identifications of prior comorbidities and liver diseases are detailed in the **Supplementary Table 1**. Drug items in each drug category are provided in **Supplementary Table 2**.

*Primary outcome and statistical analysis*

The outcome of interest was liver injury development after initial drug prescription. Univariate Cox regression was applied to identify significant predictors for shorter time to the onset of liver injury. The duration was given by the difference between initial drug prescription date to the confirmed date of liver injury or to the mortality date if the patient passed away or to the study period end date of 31^st^ December 2019 if no liver injury conditions were observed. Continuous variables were presented as median (95% confidence interval [CI] or interquartile range [IQR]) and categorical variables were presented as count (frequency). Statistical analyses were performed using RStudio software (Version: 1.1.456) or Python (Version: 3.6).

**Supplementary Table 1.** Codes for comorbidities.

| **Comorbidity** | **Codes** |
| --- | --- |
| Respiratory | 786.09, 518.81, 780.53, 137, E912, 465.9, 518.81, 518.81, 79.6, 518.81, 519.8, 780.59, 799.1, 780.57, 518.82, 480.1, 786.3, 519.8, 997.3, 165.9, 519.9, 648.91, 162.9, 162.3, 197, 162.5, 162.4, 486, 518.89, 496, 162.8, 415.1, V10.11, 518, 162.9, 11.96, 482.1, 507, 513, 11.9, 511.8, 511.1, 11.94, 516.8, 793.1, 482.4, 507, 515, 197, 11.93, 482, 482.83, 518.4, 482.3, 482.2, 415.1, 502, 518.89, 235.7, 793.1, 934.8, 516.9, 136.3, 38.49, 506, 112.4, 487, 481, 117.9, 38.2, 518, 11.95, 79.89, 518.1, 480.9, 505, 516.8, 495.9, 518.3, 11.23, 416.8, 513, 397.1, 117.3, 483, 508, 998.81, 416, 514, 861.21, 502, 934.8, 480.8, 648.93, 11.2, 492.8, 484.6, 78.5, 484.1, 516.3, 415.1, 416.9, 415, 416.9, 429.89, 415, 747.49, 745, 417, 770.7, 427.5, 416.9, 416, 416.8, 746.02, 573.8, 642.9, 416, 747.3, 747.3, 770.3, 779.8, 515, 424.3, 416, 417.8, 747.3, 747.3, 745.4, 518.81, 786.09, V12.6, 478, 748.5, 162.9, 996.84, 748.5, 748.6, V42.1, 748.5, 11.05, 162, 518, 747.42, 518.89, 748.5, 517.2 |
| Endocrine | 202.8, 200.1, 200.12, 201.9, 204, 202.88, 200.18, 196, 204.01, 785.6, 200.11, 200.13, 202.8, 785.6, 202.85, 202.81, 202.8, 202.8, 196, 196.9, 202.8, 202.82, 785.6, 202.8, 202, 202.87, V10.79, 785.6, 202.84, 202.01, 196.8, 457.1, 12.1, 785.6, V10.79, 196.5, 196.2, 238.7, 196.1, 200.14, 457.2, 238.7, V10.61, 201.9, 457, 202.8, 289.3, 245.2, 238.7, 785.6, 457.9, 785.6, 202.93, 196.9, 202.97, 757, V10.71, 288.8, 204.1, 202.83, 457.1, 289.3, 785.6, V77.9, 237.4, 239.7, 198.89, 623.5, 259.9, 200.2, V10.71 |
| Diabetes mellitus | 251.2, 362.01, 362.02, 250.4, 250.82, 790.2, 790.6, 250.5, 250.5, 250.6, 357.2, 790.2, 250, 250.4, 250.6, 250.8, 250.51, 250.5, 250.8, 250.82, 250.51, 250.51, V77.1, 250.12, 251.2, 250.12, 250.5, 250.83, 251.2, 250.41, 251.1, 250.52, 250.5, 648.81, 250.43, 250.53, 250.53, 250.81, 250.22, 250.13, 250.22, 250.83, 250.41, 250.5, 250.52, 250.52, 250.82, 253.5, V18.0, 588.1 |
| Hypertension | 401.9, 401.9, 250.82, 790.6, 401.9, 401.9, 250.82, 796.2, 402.9, 250.83, 405.99, 642.93, 642.01, 642.91, 401.9, E942.6, 405.09, 403.9, 437.2, 401, 401.1, 401, 642.33, 348.2, 779.8, 365.04, 572.3, 416, 416, 405.91, 416.8, 642.3 |
| Gastrointestinal | 153.3, 154.1, 153.9, 569.89, 154, 153.1, 578.9, 560.9, 569.3, 537.89, 558.9, 562.1, 153.6, 239, 532.3, 569.89, 532.7, 535.6, 558.9, 38.42, 569.89, 8.45, 153.2, 569.49, 79.89, 532.9, V58.11, 569, 154.1, 41.4, 537.89, 152.1, 578.9, V10.05, 787.8, 197.4, 535.5, V10.06, 9, 569.83, 569.6, 153.4, 560.9, 537.3, 41.04, 569.84, 239, 569.81, 8.8, 535, 560.9, 532, V45.89, V12.72, 532.4, V10.09, 560.81, 235.2, 38.49, 8.45, 235.2, 532.9, 569.81, 537.89, 557.9, 569.41, 997.4, 14.8, 787.99, 8.46, 535.5, 569.41, 997.4, 578.9, 569.82, 537.9, 560.1, 569.82, 557.9, 211.3, 556.9, 562, 558.9, 578.9, 536.9, 8.46, 535.6, 566, V71.9, 569.49, 564.3, V44.4, 569.89, 564.8, 8.46, 569.83, 997.4, 997.4, 997.4, 562.11, 211.2, 9.1, 211.3, 8.47, 8.5, 211.3, 569.83, 532.1, 535.61, 560, 569.83, 565.1, 619.1, 152.9, 568, 566, 569.43, 152, 8.46, 562.11, 8.61, 569.83, 569.83, 569.81, 596.1, 535.5, 151.4, 151.9, 151.5, 151.8, 151.1, 456.8, 531.7, 535.4, 531.3, V15.2, 537.89, 211.1, 531.9, V10.04, 235.2, 531, 531.4, 456.8, 535.1, 151.3, 230.2, 151.6, 535, 211.1, 536.3, 535, V10.04, 535.51, 578.9, 531.1, 535.1, 456.8, 531.5, 537.84, 535.01, 530.7, 535.1, 535.2, 535.5, 537.6, 202.83, 535.1, 531.4, 558.9, 558.9, 558, 569.85, 153, 555.1, 562.1, 562.13, 562.11, 562.12, 569.83, V76.49, 560.2, 230.4, 569.3, 154 |
| Gastrointestinal bleeding | 531, 531.2, 531.4, 531.6, 532, 532.2, 532.4, 532.6, 533, 533.2, 533.4, 533.6, 534, 534.2, 534.4, 534.6, 535.01, 535.11, 535.21, 535.31, 535.41, 535.51, 535.61, 535.71, 562.02, 562.03, 562.12, 562.13, 569.3, 569.85, 569.86, 578, 578.1, 578.9 |
| Congestive heart failure | 398.91, 402.01, 402.11, 402.91, 404.01, 404.03, 404.11, 404.13,, 404.91, 404.93, 428 |
| Hemorrhagic stroke | 431 |

**Supplementary Table 2.** Drugs prescribed.

| **ACEI**  ACEI, LISINOPRIL, PERINDOPRIL TERTBUTYLAMINE, PERINDOPRIL, RAMIPRIL, ENALAPRIL MALEATE, CAPTOPRIL, CAPTOPRIL TABLET, CAPTOPRIL, CAPTOPRIL TABLET 12.5MG--->, CAPTOPRIL, PERINDOPRIL ARGININE, PERINDOPRIL, CAPTOPRIL *FOR ORAL SOLUTION*, CAPTOPRIL, PERINDOPRIL, MALEATE, ARGININE, PERINDOPRIL, PERINDOPRIL !!!, PERINDOPRIL (E-ROOM), PERINDOPRIL (NIL), PERINDOPRIL ***, PERINDOPRIL TABLET (ACERTIL), PERINDOPRIL***, LISINOPRIL, LISINOPRIL !!!, LISINOPRIL !!!!, LISINOPRIL ***, LISINOPRIL***, LISINOPRIL*****, RAMIPRIL, ENALAPRIL MALEATE, ENALAPRIL MALEATE ***, ENALAPRIL MALEATE***, CAPTOPRIL, CAPTOPRIL !!!, CAPTOPRIL ( 6 X 25MG-->60ML), CAPTOPRIL (6.25MG>2P), CAPTOPRIL (E-RM), CAPTOPRIL (E-ROOM), CAPTOPRIL ***, CAPTOPRIL 1MG/0.2ML, CAPTOPRIL 1X25MG TO 20ML, CAPTOPRIL 2 TABS---> 40ML, CAPTOPRIL 2 X 25MG --->40ML, CAPTOPRIL 25MG X 1 --> 20ML, CAPTOPRIL 25MG X 4 TO 80ML, CAPTOPRIL 25MG X 6 > 120ML, CAPTOPRIL 25MG X 7---->70ML, CAPTOPRIL 3 X 25 MG----->60ML, CAPTOPRIL 3.125MG/0.63ML, CAPTOPRIL 3MG/0.6ML, CAPTOPRIL 6.25MG/1.25ML, CAPTOPRIL X 158  **ARB**  LOSARTAN POTASSIUM, IRBESARTAN, TELMISARTAN, CANDESARTAN CILEXETIL, CANDESARTAN, IRBESARTAN 300MG + HYDROCHLOROTHIAZ, IRBESARTAN, MICARDIS PLUS 40/12.5 (OR EQUIV), MICARDIS, MICARDIS PLUS 40/12.5 (OR EQUIV), MICARDIS, CO-DIOVAN 80/12.5 (OR EQUIV), DIOVAN, VALSARTAN, IRBESARTAN 150MG + HYDROCHLOROTHIAZ, IRBESARTAN, LOSARTAN K 50MG + HYDROCHLOROTHIAZI, LOSARTAN, LOSARTAN K 100MG + HYDROCHLOROTHIAZ, CO-DIOVAN 160/12.5 (OR EQUIV), SPARSENTAN/IRBESARTAN (CLINICAL TRI, IRBESARTAN, LOSARTAN OWN STOCK, LOSARTAN, CILEXETIL, LOSARTAN K + HYDROCHLOROTHIAZIDE, LOSARTAN POTASSIUM, LOSARTAN POTASSIUM [MEMO], IRBESARTAN + HYDROCHLOROTHIAZIDE, CO-DIOVAN (OR EQUIV), VALSARTAN |
| --- |
| **Calcium channel blockers**  AMLODIPINE (BESYLATE), OLMESARTAN/NORVASC 40MG/5MG, DILTIAZEM HCL, NIFEDIPINE, VERAPAMIL HCL, FELODIPINE, AMLODIPINE, OLMESARTAN, VERAPAMIL, AMLODIPINE BESYLATE, AMLODIPINE BESYLATE !!!, AMLODIPINE BESYLATE, AMLODIPINE BESYLATE (A4 ==>A3), AMLODIPINE BESYLATE (NORVASC), AMLODIPINE BESYLATE, AMLODIPINE BESYLATE, AMLODIPINE BESYLATE, DILTIAZEM, DILTIAZEM ( 18/2 ), DILTIAZEM HCL, DILTIAZEM HCL (E-ROOM), DILTIAZEM HCL, DILTIAZEM HCL [E-CUPBOARD], DILTIAZEM HCL S.R., DILTIAZEM HCL S.R., DILTIAZEM HCL S.R., DILTIAZEM HCL S.R., NIFEDIPINE, NIFEDIPINE !!!, NIFEDIPINE ( SL ), NIFEDIPINE (E-RM), NIFEDIPINE (E-ROOM), NIFEDIPINE (NIGHT CAB), NIFEDIPINE (NIGHT CABINET), NIFEDIPINE EXTENDED RELEASE, NIFEDIPINE S.R., NIFEDIPINE S.R., NIFEDIPINE S.R. (BAYER), NIFEDIPINE S.R. (E-RM), NIFEDIPINE S.R. (E-ROOM), NIFEDIPINE S.R., NIFEDIPINE S.R. E-CUP, NIFEDIPINE S.R. REPLACEMENT, NIFEDIPINE S.R. [E-CUPBOARD], NIFEDIPINE S.R.(E-ROOM), NIFEDIPINE S.R., NIFEDIPINE SL, VERAPAMIL, VERAPAMIL (E-ROOM), VERAPAMIL S.R., VERAPAMIL, FELODIPINE EXTENDED RELEASE, FELODIPINE EXTENDED RELEASE, FELODIPINE EXTENDED RELEASE, OLMESARTAN MEDOXOMIL TABLET, OLMESARTAN MEDOXOMIL, NIFEDIPINE, EXFORGE 5MG/80MG (OR EQUIV), EXFORGE 5MG/80MG (OR EQUIV), EXFORGE 5MG/160MG (OR EQUIV), EXFORGE 5MG/160MG (OR EQUIV), EXFORGE 10MG/160MG (OR EQUIV) , EXFORGE 10MG/160MG (OR EQUIV) |
| **Beta blockers**  ATENOLOL, BISOPROLOL FUMARATE, METOPROLOL TARTRATE, METOPROLOL, CARVEDILOL, PROPRANOLOL HCL, BISOPROLOL, METOPROLOL, PROPRANOLOL, ATENOLOL, ATENOLOL, ATENOLOL, ATENOLOL (NIL), ATENOLOL, ATENOLOL, ATENOLOL [E-CUPBOARD], ATENOLOL, ATENOLOL, ATENOLOL, METOPROLOL, METOPROLOL !!!, METOPROLOL, METOPROLOL ( 50MGX4-->160ML), METOPROLOL (100MG X4-->400ML), METOPROLOL (10ML), METOPROLOL (240ML), METOPROLOL (30ML), METOPROLOL (360ML), METOPROLOL (50MG X 4=>160ML), METOPROLOL (50MG X4-->160ML), METOPROLOL (50MGX4 --> 160ML), METOPROLOL (50MGX4-->160ML), METOPROLOL (60ML), METOPROLOL (DISPENSE 30ML-->4 DAYS), METOPROLOL (E-RM), METOPROLOL (E-ROOM), METOPROLOL 20ML, METOPROLOL, METOPROLOL, METOPROLOL {E-CUPBOARD], METOPROLOL 100MG X 1 ==> 100ML, METOPROLOL 100MG X 3 => 300ML, METOPROLOL 100MGX1-->100ML, METOPROLOL 1MG/ML X 250ML, METOPROLOL 1MG/ML X 300ML, METOPROLOL 4X 50MG==>160ML, METOPROLOL 50MG 1 TAB --> 50ML, METOPROLOL 50MG 1 TAB---->50ML, METOPROLOL 50MG 1TAB --> 50ML, METOPROLOL 50MG X 1-->40ML, METOPROLOL 50MG X 1TAB------>50ML, METOPROLOL 50MG X 4 ==> 160ML, METOPROLOL 50MG X 4 ==>160ML, METOPROLOL 50MG X 4 --> 160ML, METOPROLOL 50MG X 4 ---> 160ML, METOPROLOL 50MG X 4 ->160ML, METOPROLOL 50MG X 4 -->160ML, METOPROLOL 50MG X 4 S==>160ML, METOPROLOL 50MG X 4`S-->160ML, METOPROLOL 50MG X 4==> 160ML, METOPROLOL 50MG X 4---> 160ML, METOPROLOL 50MG X 4-->160ML, METOPROLOL 50MG X 6 ---> 240ML, METOPROLOL 50MG X-->160ML, METOPROLOL 50MG X4 --->160ML, METOPROLOL 50MG X4->160ML, METOPROLOL 50MG X4-->160ML, METOPROLOL 50MG X4--->160ML, METOPROLOL 50MG/TAB (1TAB---->50ML), METOPROLOL 50MGX 4==>160ML, METOPROLOL 50MGX1-->40ML, METOPROLOL 50MGX2S-->100ML, METOPROLOL 50MGX4 -->160ML, METOPROLOL 50MGX4==>160ML, METOPROLOL 50MGX4----> 160ML, METOPROLOL 50MGX4->160ML, METOPROLOL 50MGX4--->160ML, METOPROLOL 50MGX4-160ML, METOPROLOL 7 X 50MG >35ML, METOPROLOL C.R., METOPROLOL S.R., METOPROLOL SYRUP 1X50MG--> 50ML, METOPROLOL SYRUP50MGX3-->120ML, METOPROLOL X 360ML, METOPROLOL X 60ML, METOPROLOL X300ML, METOPROLOL X360ML, METOPROLOL X4=>160ML, METOPROLOL(190ML), METOPROLOL(320ML), METOPROLOL(400), METOPROLOL(90ML), METOPROLOL(E-ROOM), METOPROLOL***, METOPROLOL50MGX4->160ML, METOPROLOLN 50MG X4 ---> 160ML, METOPROLOLX240ML, CARVEDILOL, CARVEDILOL !!!, CARVEDILOL, CARVEDILOL, PROPRANOLOL HCL, PROPRANOLOL HCL (40MG X 10->400ML), PROPRANOLOL HCL (E-ROOM), PROPRANOLOL HCL, PROPRANOLOL HCL, PROPRANOLOL HCL 40MG X 15 -> 600ML, PROPRANOLOL HCL 40MGX10>400ML, PROPRANOLOL HCL S.R., PROPRANOLOL HCL |
| **Antihypertensive agents**  METHYLDOPA, DOXAZOSIN (MESYLATE) GITS, TERAZOSIN HCL, METHYLDOPA, METHYLDOPA, METHYLDOPA (250MG X 10 -->100ML), METHYLDOPA (E-ROOM), METHYLDOPA (HOME MED), METHYLDOPA, METHYLDOPA, METHYLDOPA 10 X 250MG TO 100ML, METHYLDOPA 250MG X 40 ---->2000ML, METHYLDOPA 250MG X 40--->100ML, METHYLDOPA 250MG X 6 --> 60ML, METHYLDOPA 250MG X 6--->60ML, METHYLDOPA 250MG X21 TO 105ML, METHYLDOPA 250MGX40 => 200ML, METHYLDOPA 28 X 250MG TO 140ML, DOXAZOSIN MESYLATE, DOXAZOSIN MESYLATE, DOXAZOSIN MESYLATE (NIL), DOXAZOSIN MESYLATE, DOXAZOSIN MESYLATE, DOXAZOSIN MESYLATE GITS, DOXAZOSIN MESYLATE GITS, DOXAZOSIN MESYLATE GITS, TERAZOSIN HCL, TERAZOSIN HCL !!!, TERAZOSIN HCL, TERAZOSIN HCL (E-RM), TERAZOSIN HCL, TERAZOSIN HCL(E-RM), TERAZOSIN HCL(E-ROOM), PRAZOSIN HCL, PRAZOSIN HCL, PRAZOSIN HCL, PRAZOSIN HCL PRAZOSIN HCL (E-ROOM), PRAZOSIN HCL (HOME MED), PRAZOSIN HCL (NIL), PRAZOSIN HCL, PRAZOSIN HCL 1MG X11-->55ML  AMLODIPINE (BESYLATE), OLMESARTAN/NORVASC 40MG/5MG, DILTIAZEM HCL, NIFEDIPINE, VERAPAMIL HCL, FELODIPINE, AMLODIPINE, OLMESARTAN, VERAPAMIL, AMLODIPINE BESYLATE, AMLODIPINE BESYLATE, AMLODIPINE BESYLATE, AMLODIPINE BESYLATE (A4 ==>A3), AMLODIPINE BESYLATE (NORVASC), AMLODIPINE BESYLATE , AMLODIPINE BESYLATE , AMLODIPINE BESYLATE, DILTIAZEM, DILTIAZEM ( 18/2 ), DILTIAZEM HCL, DILTIAZEM HCL (E-ROOM), DILTIAZEM HCL, DILTIAZEM HCL [E-CUPBOARD], DILTIAZEM HCL S.R., DILTIAZEM HCL S.R., DILTIAZEM HCL S.R., DILTIAZEM HCL S.R., NIFEDIPINE, NIFEDIPINE !!!, NIFEDIPINE ( SL ), NIFEDIPINE (E-RM), NIFEDIPINE (E-ROOM), NIFEDIPINE (NIGHT CAB), NIFEDIPINE (NIGHT CABINET), NIFEDIPINE EXTENDED RELEASE, NIFEDIPINE S.R., NIFEDIPINE S.R., NIFEDIPINE S.R. (BAYER), NIFEDIPINE S.R. (E-RM), NIFEDIPINE S.R. (E-ROOM), NIFEDIPINE S.R., NIFEDIPINE S.R. E-CUP, NIFEDIPINE S.R. *REPLACEMENT*, NIFEDIPINE S.R. [E-CUPBOARD], NIFEDIPINE S.R.(E-ROOM), NIFEDIPINE S.R., NIFEDIPINE SL, VERAPAMIL, VERAPAMIL (E-ROOM), VERAPAMIL S.R., VERAPAMIL, FELODIPINE EXTENDED RELEASE, FELODIPINE EXTENDED RELEASE, FELODIPINE EXTENDED RELEASE |
| **Diuretics for heart failure**  SPIRONOLACTONE, FRUSEMIDE (FUROSEMIDE), METOLAZONE, SPIRONOLACTONE, SPIRONOLACTONE, SPIRONOLACTONE *E-CUP*, FRUSEMIDE, FRUSEMIDE, FRUSEMIDE (500MG X 1 > 125ML), FRUSEMIDE FORTE, FRUSEMIDE PAEDIATRIC, METOLAZONE, AMIODARONE HCL, AMIODARONE HCL !!!, AMIODARONE HCL (200MG X 8 > 40ML), AMIODARONE HCL, AMIODARONE HCL, AMIODARONE HCL 12 X 200MG TO 60ML, AMIODARONE HCL 14X200MG TO 70ML, AMIODARONE HCL 200MG X 14---->70ML, AMIODARONE HCL 200MGX7->35ML |
| **Nitrates**  ISOSORBIDE DINITRATE, ISOSORBIDE, DINITRATE, ISOSORBIDE DINITRATE, ISOSORBIDE DINITRATE !!!, ISOSORBIDE DINITRATE (E-ROOM), ISOSORBIDE DINITRATE, ISOSORBIDE DINITRATE, ISOSORBIDE DINITRATE [E-CUPBOARD], ISOSORBIDE DINITRATE 168TAB, ISOSORBIDE DINITRATE 210, ISOSORBIDE DINITRATE 504, ISOSORBIDE DINITRATE S.R., ISOSORBIDE DINITRATE S.R., ISOSORBIDE DINITRATE S.R. (E-RM), ISOSORBIDE DINITRATE S.R. (E-ROOM), ISOSORBIDE DINITRATE S.R. (ISOKET), ISOSORBIDE DINITRATE, ISOSORBIDE DINITRATE, ISOSORBIDE MONONITRATE, ISOSORBIDE MONONITRATE !!, ISOSORBIDE MONONITRATE !!!, ISOSORBIDE MONONITRATE (E-ROOM), ISOSORBIDE MONONITRATE, ISOSORBIDE MONONITRATE C.R., ISOSORBIDE MONONITRATE C.R., ISOSORBIDE MONONITRATE C.R. !!!!!, ISOSORBIDE MONONITRATE C.R., ISOSORBIDE MONONITRATE C.R., ISOSORBIDE MONONITRATE S.R., ISOSORBIDE MONONITRATE, ISOSORBIDE S.R. (=ISOKET RETARD), GLYCERYL TRINITRATE, GLYCERYL TRINITRATE (NO ALCOHOL), GLYCERYL TRINITRATE (NO ALCOHOL), GLYCERYL TRINITRATE 200 DOSE UNIT, GLYCERYL TRINITRATE 200DOSE(S), GLYCERYL TRINITRATE S.R., GLYCERYL TRINITRATE S.R. PAT (E-CUP, GLYCERYL TRINITRATE S.R. PATCH, GLYCERYL TRINITRATE S.R. PATCH(TIS), GLYCERYL TRINITRATE TRANSDERMAL, GLYCERYL TRINITRATE(HOME MED), GLYCERYL TRINITRATE(MAINTENANCE PK)  **Statins and Fibrates**  SIMVASTATIN, FLUVASTATIN, ATORVASTATIN, GEMFIBROZIL |
| **Antihyperlipidemic/Lipid-lowering drugs**  Lovastatin, ATORVASTATIN, ATORVASTATIN !!!!, AMLODIPINE BESYLATE, AMLODIPINE BESYLATE*****, SIMVASTATIN, SIMVASTATIN **, SIMVASTATIN**********, ATORVASTATIN, ATORVASTATIN !!!!, SIMVASTATIN, SIMVASTATIN **, SIMVASTATIN**********, [OWN] ATORVASTATIN (CALCIUM), ATOR01 ATORVASTATIN (CALCIUM) TABLET 10MG (LIPITOR), ATOR02 ATORVASTATIN (CALCIUM) TABLET 20MG (LIPITOR), ATOR03 ATORVASTATIN (CALCIUM) TABLET 40MG (LIPITOR), ATOR04 ATORVASTATIN (CALCIUM) TABLET 80MG (LIPITOR), ATORVASTATIN, ATORVASTATIN !!!!, ATORVASTATIN (CALCIUM), ATORVASTATIN (CALCIUM) ##, ATORVASTATIN (CALCIUM) (OWN), ATORVASTATIN (CALCIUM) ***, ATORVASTATIN (CALCIUM)##, ATORVASTATIN (CALCIUM)***, FLUV02 FLUVASTATIN (SODIUM) CAPSULE 20MG (LESCOL), FLUV03 FLUVASTATIN (SODIUM) CAPSULE 40MG (LESCOL), FLUV05 FLUVASTATIN(SODIUM)EXTENDED RELEASE TABLET 80MG (LESCOL XL), Pitavastatin, PRAVASTATIN SODIUM, PRAVASTATIN, Pravachol, ROSU01 ROSUVASTATIN (CALCIUM) TABLET 10MG (CRESTOR), ROSU02 ROSUVASTATIN (CALCIUM) TABLET 20MG (CRESTOR), ROSUVASTATIN (CALCIUM), SIMV01 SIMVASTATIN TABLET 10MG (ZOCOR), SIMV02 SIMVASTATIN TABLET 20MG (ZOCOR), SIMV04 SIMVASTATIN TABLET 40MG (ZOCOR), SIMVASTATIN, SIMVASTATIN (OS), SIMVASTATIN **, SIMVASTATIN ***, SIMVASTATIN [OWN MED], SIMVASTATIN***, SIMVASTATIN*********, SIMVASTATIN**********, SIMVASTATIN**17/5**, SIMVASTATIN**23/12**, SIMVASTATIN**29/8***, [OWN] EZETIMIBE, EZETIMIBE, EZET01 EZETIMIBE TABLET 10MG (EZETROL), EZETIMIBE, EZETIMIBE ## |
